# Supplementary material for: Are carbon emissions trading and green financial instruments synergistic? -Comprehensive quantitative research based on content analysis
Source: PLoS One. 2024 Mar 7;19(3):e0298601. doi: 10.1371/journal.pone.0298601 (PMC10919601; doi:10.1371/journal.pone.0298601)
Supplement: S1 Data — (ZIP) [file pone.0298601.s001.zip › Data/地级市Z_F工作报告.zip!/╦─┤¿/╣π╘¬╩╨/└┤╫╘╣1⁄2╓┌║┼í╛┬φ┐╦╩2╛▌═°í┐_2017.docx]

各位代表：

现在，我代表市人民政府向大会报告工作，请予审查，并请各位政协委员提出意见。

2016年工作回顾

过去一年，面对持续加大的经济下行压力和艰巨繁重的改革发展稳定任务，在省委、省政府和市委的坚强领导下，在市人大、市政协的监督支持下，我们紧紧依靠全市人民，全面实施“三个一、三个三”战略体系，采取一系列稳增长、促发展的政策措施，全市经济社会持续健康发展。全年实现地区生产总值660.01亿元、增长8%，增速居全省第9位、川东北经济区第1位，其中一二三产业分别增长3.9%、8.9%、8.7%，全社会固定资产投资618.35亿元、增长6%，增速为近6年最高，社会消费品零售总额331.07亿元、增长11.6%，地方一般公共预算收入40.57亿元、增长2.5%，城乡居民人均可支配收入分别达到25762元、9819元，分别增长9%、9.8%，增速居全省第1位、第4位，居民消费价格上涨1.9%，圆满完成市人代会确定的目标任务，实现“十三五”良好开局。

一、集中力量精准发力，“三大主战场”战绩喜人

脱贫攻坚首战告捷。出台33条措施，统筹各层级931个部门单位帮扶力量，超常推进脱贫攻坚，204个贫困村、7.04万贫困人口达到退出和脱贫标准，超额完成省下达目标任务。突出产业和就业扶贫，贫困户人均纯收入5497元。资助建档立卡贫困学生8.54万人次，无学生因贫辍学。全面落实贫困人口就医“十免四补助”和八个100%政策，县域内就医个人支出控制在10%以内。易地扶贫搬迁8160户、2.67万人，改造贫困户危房8107户。创新农村产权扶贫和金融扶贫模式，发放扶贫小额信贷12.07亿元。通村硬化路、村卫生室、村文化室、通信网络覆盖全部达标。旺苍县通过国家省际间交叉考核，苍溪县通过国家第三方评估。全国县级“十三五”脱贫攻坚规划编制工作会、全省就业扶贫现场推进会等在广元召开，健康扶贫“四大工程”、脱贫奔康“六化”行动等做法分别在全国、全省推广。

项目投资增速加快。深入开展“项目年”活动，精准对接国家重点投向，全力争取国家专项建设基金，深化重大项目“四个一批”生成机制、“四个一”推进机制，强化土地、资金等要素保障，向上争取资金68.92亿元，是上年的2.4倍，161个省市重点项目完成投资302.22亿元，新开工项目投资占全社会固定资产投资的50.7%。西二环、凉水泉风电场等41个重点项目开工建设，稀土铝合金电缆、主城区生活垃圾焚烧发电等24个重点项目竣工，西成客专广元段等67个重点项目加快推进，北二环东延线等35个重点项目前期工作有序开展。民间投资增长9.9%，增速居全省第5位。大力推行PPP模式，签约入库项目5个、总投资36.36亿元，入选国家第三批示范项目2个，个数居全省第2位。

三次产业加快发展。坚持工业强市，全力推动工业经济稳增长，规模以上工业增加值增长9.7%，增速居全省第9位。五大特色优势产业占规模以上工业总产值的66.6%，战略性新兴产业产值增长10.2%。规模以上工业企业主营业务收入增长11.3%、利润总额增长21.1%。完成工业投资195.6亿元、技改投资136.3亿元，广元机电产业园、2亿安时锂离子电池等重点项目加快建设。新进规企业42户，对规模以上工业增长的贡献率达49.2%。实行投资项目、工业企业、民营企业、外贸出口企业问题会商制度，“一企一策”解决重点企业困难，提升对企服务水平。协调争取直购电优惠政策，实现启明星铝业脱困、中哲新材料复产；推动攀成钢焦化与川能投战略重组，协调铁路货运价格下降55%，实现旺苍煤焦化产业链全面盘活；协调解决融资难题，推动亿明药业新生产线全面竣工；组织开展食品饮料产品系列推介活动，引导企业利用“京东广元馆”等电子商务平台强化网上销售，“广元造”产品市场影响力进一步扩大；常态化开展银企对接活动，用好工业企业贷款风险补偿基金，中小微工业企业累计获得贷款7.89亿元、增长124.1%。加大工业园区基础设施及配套建设投入，园区承载能力进一步增强。建筑业增加值增长8.7%。

加快发展以商贸物流、康养旅游为重点的现代服务业。图腾·海博春天购物广场、广元物流港上西园区等项目建成投运，农村商贸网点和配送体系逐步健全，社区服务、体育健身、文化娱乐等服务业态加快发展。苍溪县、利州区分别成功申报创建国家级、省级电子商务进农村综合示范县。银行业金融机构存贷款余额分别增长14.8%、14.9%。推进中国生态康养旅游名市建设，突出全域旅游带动，成功创建全国旅游标准化示范城市，苍溪县被评为省级旅游扶贫示范县，全市新增2个国家4A级旅游景区。中青城投昭化古城、华侨城剑门关景区、环球雅途广元旅游目的地等重大项目成功签约。全年接待游客3792.07万人次、旅游总收入264.22亿元，分别增长16.6%、27.6%。

大力培育“6+1”特色产业，新建现代农业园区7个、提升7个，新增特色产业基地18万亩。突破性发展肉牛羊产业，出栏肉牛7.25万头、肉羊33.73万只。生态渔业发展迈出实质性步伐。粮食产量实现十连增。实现劳务总收入170.62亿元。“1+3”新型农业经营体系加快构建，推进新型农业经营主体与农民建立紧密利益联结机制，新培育销售过亿元龙头企业13家、农民合作社764个、家庭农场1268个、农业社会化服务超市185家，创建省级示范社（场）42家。与央视农业频道合作，强化特色优势农产品品牌打造与营销，剑门关土鸡销售增长30%，特色农产品出口增长43.4%。

二、创新改革不断深化，对外开放合作持续扩大

科技创新取得新成果。推进产学研用协同创新，引进各类高层次人才259名，签订猕猴桃深加工等产学研合作协议45项，新建企业工程技术研究中心、科技孵化器、院士（专家）工作站等各类创新平台37个。申请专利1585件，取得重大科技成果42项，实施科技成果转化项目148项，新培育高新技术企业25家。实现高新技术产业产值171亿元、增长12%，科技进步对经济增长的贡献率达47%。

重点领域改革和供给侧结构性改革取得新进展。出台专项改革方案52个，落实全面创新改革试验9张清单，实施国家、省级改革试点36项。基本完成农村产权“七权同确”，推进农业供给侧结构性改革，建成农村土地承包经营权确权登记和交易管理平台，耕地、林地经营权流转率分别达到31%、11.6%。全省深化农村改革工作现场会在广元召开。“五证合一”、“三证整合”商事制度改革稳步推进，在全省率先全面推行“一窗进出”并联审批。党政机关公务用车制度改革基本完成。出台供给侧结构性改革“1+5”方案，压减煤炭产能39万吨，房地产去库存97万平方米，专项清理金融机构收费，多方争取降低要素价格，降低企业成本5.6亿元。

对外开放合作取得新成效。加大投资促进力度，引进到位市外资金458亿元、增长11.7%。密切与国外、境外交流合作，在食品产业、生态产业、职业技能培训等方面签订合作协议。深入推进与长三角、珠三角、环渤海地区、西北地区和川陕甘毗邻地区的交流合作，签订合作协议87个、签约资金198.63亿元。充分利用西博会、农博会、女儿节等节会展会，大力开展项目推介活动。成功举办川陕革命老区振兴发展论坛、九广合作30周年、浙广合作20周年系列活动。在珠三角、华北、华东地区和新疆召开返乡创业回家发展座谈会，新增农民工和旅外企业家返乡创业3360人，新增经济实体3310个。

三、城乡统筹步伐加快，生态建设扎实推进

新型城镇化有序推进。坚持规划引领，启动新一轮城市总体规划修编和中心城区控规动态维护，完成7个镇21个村“多规合一”和200个新村聚居点规划编制。新增城市建成区5平方公里，城镇化率提高1.57个百分点，增幅居全省第3位。则天路延伸段、广元至昭化城区快速通道建成通车，国道108线陵江至宝轮段改线、严家湾隧道等项目加快推进，市城区交通体系不断完善。小城镇建设提质增效，统筹城乡发展综合示范镇建设扎实推进，就地就近吸纳转移人口3.2万人。

幸福美丽新村加快建设。新建幸福美丽新村299个、聚居点356个、“1+6”村级公共服务活动中心255个，新改建农村公路1550公里，基础设施和公共服务进一步向农村延伸。整治塘堰430座，新建和整治渠道510公里，新增有效灌面2.1万亩，新解决10万农村人口安全饮水问题。创建省级“四好村”67个。全省农田水利基本建设现场会在广元召开。

绿色发展迈出新步伐。积极开展国家生态文明先行示范区创建，持续推进低碳城市建设,低碳发展经验在中欧低碳城市会议、全国低碳试点城市总结评估会议上交流。启动实施大规模绿化全川广元行动，森林覆盖率达到55.78%。建成柏林湖国家湿地公园。在全省率先开展污染防治“三大战役”。“无霾城市”建设扎实推进，沿街烤火、烧烤、祭祀、垃圾和秸秆焚烧治理成效明显，市城区烟雾源头有效管控，工业烟粉尘排放达标率98%，空气质量居全省第2位。实施嘉陵江、白龙江流域广元段污染综合治理和生态环境保护项目，全面规范三江新区范围内河道采砂，拆除“两湖”违规钓鱼平台，全面启动市城区黑臭水体整治，主要流域水质稳定达到Ⅱ类标准，县级以上城市集中式饮用水水源地水质稳定达标。农村生活垃圾集中处理、饲料清洁化行动有力推进，农村面源污染治理进一步加强。单位GDP能耗下降5.09%，总量减排任务全面完成。国家卫生城市创建成果进一步巩固，市容市貌明显改善。

四、民生福祉持续增进，社会事业协调发展

民生保障持续改善。全面完成十项民生工程和30件民生大事，民生支出占一般公共预算支出的68.5%。扶持创业1729人，城镇新增就业4.17万人，城镇登记失业率3.84%。城镇五项社会保险参保166.8万人，城乡居民基本养老保险覆盖114.28万人，新农合参合212.28万人。城乡居民最低生活保障标准、企业退休人员基本养老金、孤儿分散和集中供养标准提高。新开工改造城镇危旧房棚户区5871套，货币化安置1883户，定向供应农民工公租房754套。改造农村危房1.1万户。

社会事业全面发展。普惠性幼儿园覆盖率87.8%，适龄残疾儿童少年义务教育入学率90%。利州区、旺苍县义务教育均衡发展分别通过国家、省级督导评估验收。高中阶段教育质量全面提升，全市高考重点本科上线人数增长46.8％。中职学生就业率98.5%。分级诊疗全域实施，县域内就诊率逐步提高，异地就医即时结算范围扩大。被确定为国家级医养结合试点市。“智慧卫生计生”项目基本建成，居民健康卡实现首发，城乡居民健康档案电子建档率96.46%。实施全面二孩政策，妇幼健康服务水平不断提升。推进文化惠民工程，建成234个村文化室、144个村文化院坝，成功创建“中华诗词之市”。千佛崖保护修复工程全面完成。积极开展群众体育健身活动，成功举办省青少年体育锦标赛等体育赛事，大力推进省运会筹备工作。省级文明城市创建通过实地考察。第三次全国农业普查工作受到国务院农普办、国家统计局肯定。审计、住房公积金、防震减灾、测绘、气象、水文、工会、青少年、妇女儿童、老龄、残疾人、红十字会、保险、慈善、地方志、外事侨务、档案、科普、爱国卫生等工作有效开展。

五、依法治市深入推进，治理水平稳步提高

社会治理不断创新。强化社会治安综合治理，网格化服务管理实现全覆盖，网上公安局标准化建设经验在全国推广。建立川陕甘三省五市边界人民调解协作机制。司法责任制改革顺利推进，公共法律服务体系更加完善。“七五”普法全面启动，“法治政务”建设和“法律七进”活动深入推进。深化“五建五强”群众工作体系，基层群众自治制度不断完善，和谐社区创建有效推进。社会组织管理不断加强，61个行业协会商会与行政机关脱钩。

坚决守住“三条底线”。严格落实安全生产责任，持续深化重点行业领域专项整治，切实整改重大安全隐患，无重特大安全生产事故发生。严格规范食品药品过程监管，重大食品药品安全实现“零事故”。接访下访、包案化解形成常态，铁路建设施工、关闭煤矿职工安置等一批重大涉稳问题有效解决，信访总量连续6年下降。妥善处理白龙湖沉船等突发事件。依法打击违法犯罪活动，社会保持和谐稳定，群众安全感满意度第三方测评居全省第1位，是全省唯一、全国30个最安全城市之一。被评为全国人民防空工作先进集体，成功争取海军一艘护卫舰正式命名为“广元舰”，双拥模范城创建实现“五连冠”。国家安全、保密、消防、对台、民族宗教、防邪、国防动员、民兵预备役等工作全面加强。

六、政府职能加快转变，自身建设不断加强

深入推进“放管服”改革，全面清理、动态调整39个市级部门行政权力责任清单，取消、下放、委托下放行政审批项目和公共服务事项139项，清理项目审批前置条件802项、投资项目报建审批事项65项。经开区行政审批权相对集中改革试点取得初步成效。健全政府法律顾问制度，完善行政应诉工作规程，严格重大行政决策法定程序。修订完善政府工作规则和议事规则。深化政府信息公开，行政权力依法规范公开运行事项办结率91.75%。完成人大代表建议、政协委员提案办理，满意率100%。认真开展“两学一做”学习教育，强化作风效能建设，创办“阳光问政”，持续优化发展环境。强力正风反腐，查处腐败案件990件，移送司法机关23人，廉政建设社会评价居全省第3位。

各位代表，过去一年取得的成绩，是省委、省政府坚强领导、深切关怀的结果，是市委加强领导、科学决策的结果，是市人大、市政协有效监督、大力支持的结果，是全市广大干部群众辛勤工作、共同努力的结果。在此，我代表市人民政府，向全市人民，向人大代表、政协委员，向各民主党派、工商联、无党派人士、各人民团体和社会各界人士，向驻广解放军、武警官兵、政法干警和民兵预备役人员，向所有关心支持广元发展的朋友们，表示衷心的感谢!

我们也清醒地认识到，我市经济社会发展中还存在不少困难和问题。项目投资缺少大项目、好项目，特别是重大产业项目；实体经济面临诸多困难，一些要素成本较高，部分企业资金缺乏，部分行业指标难以维持高增长；新的财税增长点不多，增收难度较大；县域经济实力不强，脱贫攻坚任务仍然艰巨；高层次人才缺乏，自主创新能力不强；安全生产形势不容乐观，个别环境指标有所下降；政府系统少数人员服务意识不强，庸政懒政怠政现象依然存在。

2017年主要目标任务和重点工作

2017年是实施“十三五”规划的重要一年，是供给侧结构性改革的深化之年。政府工作的总体要求是：全面贯彻党的十八大和十八届三中、四中、五中、六中全会及省委十届六次、七次、八次、九次全会精神，全面贯彻落实中央和省委经济工作会议部署，全面落实市第七次党代会、市委七届二次全会和市委经济工作会议精神，按照“五位一体”总体布局和“四个全面”战略布局要求，始终保持专注发展战略定力，切实践行新发展理念，深入落实省委治蜀兴川方略，坚定不移实施“三个一、三个三”战略体系，坚持稳中求进、加快发展工作总基调，坚持以做大总量和提升质量效益为中心，坚持以推进供给侧结构性改革为主线，全面做好稳增长、促改革、调结构、惠民生、防风险各项工作，推动经济持续较快发展，保持社会和谐稳定，在从整体连片贫困到同步全面小康跨越上迈出新步伐。

主要预期目标建议：地区生产总值增长8%，规模以上工业增加值增长10%，全社会固定资产投资增长13%，社会消费品零售总额增长11%，地方一般公共预算收入增长8%，城乡居民人均可支配收入分别增长9%、10%，居民消费价格涨幅控制在3%以内，城镇登记失业率控制在4%以内，城镇化率提高1.5个百分点，完成节能减排目标任务。

实现上述目标，今年重点抓好以下九个方面的工作：

一、聚焦脱贫攻坚，提升精准扶贫精准脱贫质效

咬紧脱贫攻坚任务。大力实施“五个一批”和“25个扶贫专项”年度计划，强力推进脱贫奔康“六化”行动，确保利州区率先摘帽、255个贫困村退出、6.78万贫困人口脱贫。突出抓好产业和就业扶贫，培育长短结合、特色突出、效益良好的增收产业，强化新型农业经营主体带动，做好贫困户技能培训和转移就业，推行入股分红、合作经营、资产收益等模式，确保每个计划脱贫户至少有1个增收项目或1人就业。发展壮大集体经济。加快改善贫困地区生产生活条件，完善交通、水利、电力、通信等基础设施，解决进村入户“最后一公里”问题。抓好住房安全建设，加快推进易地扶贫搬迁、新村建设、农村危旧房改造。抓好医疗、教育扶贫，推动基层医疗、教育机构软硬件达标，落实好医疗扶贫“十免四补助”、学前教育和义务教育等扶持政策，用好教育、卫生扶贫救助基金，解决好因病致贫返贫、看病贵、因贫辍学等问题。

创新工作推进机制。充分发挥脱贫攻坚组织指挥体系作用，从严落实主体责任和“六个一”帮扶机制，实行帮扶清单管理，发挥部门职能，健全行业分工、协同配合推动脱贫攻坚工作机制。推动国省定点扶贫、浙江扶贫协作，鼓励支持社会力量参与扶贫。落实支持龙头企业带动脱贫攻坚的政策措施。创新金融扶贫，加大项目资金整合、金融资金运用、社会资金投入力度，健全脱贫攻坚投入保障机制。建立完善脱贫对象跟踪监测、动态管理和返贫问责机制，巩固深化脱贫成果。办好农民夜校，加强思想道德和感恩教育，激发群众内生动力，引导群众养成好习惯、形成好风气。

二、着力项目投资，拉动经济稳定增长

强化项目储备和争取引进。精准对接重大政策机遇，提高项目谋划水平，在重大基础设施、新型工业、现代农业、新型城镇化、生态康养旅游、现代物流、社会事业等领域储备一批重大项目，确保常态化储备1万亿元以上。着眼加快即期投资增长、稳定未来投资预期，向上争取一批项目，力争中央基金项目实现新的大幅增长。改革招商体制，强化奖惩激励，确保招商引资较大幅度增长，全年引进到位市外资金527亿元以上、增长15%以上。鼓励民间投资，确保民间投资较快增长。发展创业空间和孵化基地，培育一批项目。

强化重大项目推进。深入实施“项目年”，落实项目推进六大机制。抓好季度集中开工，力争全年新开工项目完成投资400亿元以上。抓好200个重点建设项目，完成投资335亿元以上。推动国道108线瓷窑铺至沙溪坝公路改建、电容触摸屏生产线等25个重点项目竣工投运，七盘关国际石材城、红星美凯龙综合体等66个重点项目加快建设，石盘产城融合园、装配式混凝土构件生产线等109个重点项目开工建设，力争绵广高速复线、广巴铁路扩能改造、动车所、中欧班列广元组货基地等重点项目前期工作有实质性进展。

强化项目要素保障。创新投融资体制，拓宽项目融资渠道。加快组建各类基金，推进债券发行。强化银行金融机构存贷比考核，确保对全市重大基础设施、重大产业、战略性新兴产业和军民融合产业项目贷款持续增长。抓好中央投资项目资金管理，盘活财政存量资金，提升资金使用效率。积极争取新增建设用地计划，精细化管理用地指标，开展闲置土地清理，盘活用好存量建设用地，加快征地拆迁进度和建设用地审批，落实好差别化用地政策，千方百计保障发展用地。抓好煤、电、油、气、运等要素保障。加大经济发展环境治理力度，优化项目施工环境。

三、狠抓产业发展，加快转型升级步伐

做强做优工业经济。突出工业主导地位，力争工业发展取得新进展。加快娃哈哈第二生产基地、日航机电等重大工业项目建设，完成工业投资220亿元以上、技改投资150亿元以上。培育新进规企业40家以上。坚持企业问题会商制度，进一步改善实体经济政务服务。完善市场化建园模式，新增工业园区面积4000亩以上。建成云计算大数据中心，促进信息化工业化深度融合。实施品牌战略，打造一批国省名优品牌。强化产品营销，提高“广元造”产品市场占有率。

加快发展现代服务业。大力发展电子商务、现代物流、现代金融、科技服务、健康养生等新兴先导型服务业，加快发展生产性服务业。加快川北国际商贸物流港、川北国际汽车城、农产品交易中心迁建工程等项目建设。着力培育重点企业，打造重点品牌。培育新消费热点，促进电商、信息、文化等消费。提升市场拓展“三大活动”，办好女儿节商品展销会、秦巴山区年货节等展会，政府采购以及政府性工程建设所需货物、服务，在同等条件下优先使用“广元造”产品。继续开展食品饮料及农产品推介活动，做强广元特色餐饮，培育“广元味道”。抓好“放心舒心消费城市”创建。

突破性发展生态康养旅游业。全面启动全域旅游示范市创建，积极推进中国生态康养旅游名市建设，打造大蜀道国际旅游目的地。推进国家旅游业改革创新先行示范区、国家级医养结合试点市以及剑阁县、青川县全域旅游示范区建设。加快推进中青城投、华侨城、广元康养示范产业园等项目落地开工，启动创建米仓山国家5A级旅游景区、唐家河和昭化古城国家级旅游度假区、曾家山中国农业公园及农旅融合示范区、天曌山国家级康养旅游示范基地，大力开发森林、温泉、中医药、体育、健身休闲等一批康养旅游产品，积极发展特色康养旅游商品。组建文旅集团，形成集建设、管理、融资、营销于一体的生态康养旅游发展平台。办好生态康养旅游高峰论坛、蜀道文化旅游节和广元女儿节。力争全年接待游客4255万人次，实现旅游总收入325亿元。

积极推进现代农业。推动现代农林牧业重点县建设，做强“6+1”特色产业，壮大特色优质农产品规模，发展精深加工。突破性发展肉牛羊产业，大力发展生态渔业，健全剑门关土鸡产销体系。新建现代农业园区7个，大力培育种养大户、家庭农场、农民合作社和产业化龙头企业，做强“7+3”特色农业品牌。鼓励新型经营主体探索融合发展模式，与农民建立更加紧密的利益联结机制。大力推进农村电商发展。

四、深化改革创新开放，激发内生动力与活力

抓好供给侧结构性改革。深入推进“三去一降一补”。引导煤炭企业化解过剩产能，压减普通水泥产能，支持电解铝延链升级。推动房地产业持续平稳健康发展，进一步减少商品住房库存。优化信贷投向，加大对小微企业的支持力度。大力发展直接融资，引导和支持企业通过股权融资、银行间市场融资，实现各类直接融资超过80亿元。落实减税降费政策，积极争取直购电、直供气、富余电量消纳等政策，协调降低铁路运价，进一步降低企业要素和物流成本。持续推进“一提一创一培”，加强质量强市建设，开展主导产业、重点产品对标提升和品牌创建行动。推进农业供给侧结构性改革，把增加绿色优质农产品供给放在突出位置，狠抓农业科技创新、农产品标准化生产、品牌创建、质量安全监管。围绕薄弱环节和滞后领域，增加公共产品和公共服务供给。

深化重点领域改革。推行农村集体资产股份合作制改革，推进承包土地“三权分置”和各项农村改革试点。推进国有企业管理体制改革，完善企业经营机制。深化财税体制改革，硬化预算约束，坚持一本预算管一年，全面公开预决算信息。力争完成农村信用社改制组建农商银行。深化商事制度改革。实现“双随机、一公开”监管全覆盖，加强和完善事中事后监管。推进生态文明体制改革，完善生态补偿机制。抓好督察落实，推动已出台改革方案落地见效。

推进科技创新。深化市校合作、厅市会商，推进产学研用协同创新，提高企业自主创新能力。新签订一批产学研合作协议，新建一批创新创业平台，实施好重点研发计划，支持专利申请和科技成果转化，实现高新技术产业产值190亿元，科技进步对经济增长的贡献率提高到48%以上。创新人才引进、培育、使用、激励机制，吸引更多科技人才、高层次人才、青年大学生、乡土人才进入创新创业主战场。

深化对外开放合作。实施大开放大合作战略，建设国家内陆开放合作示范市。加强与国外、境外交流合作，推动外贸出口大幅度增长。扩大与长三角、珠三角、环渤海地区的交流合作，以广元籍企业家聚集地区为重点，加大返乡创业服务和承接产业转移力度。密切与成渝经济区经济联系，加强与川陕革命老区其他城市及毗邻城市合作，主动对接区域产业分工。充分利用贫困地区企业上市支持政策，借力中外知名企业四川行、科博会等重大投资促进活动，切实开展对外交流合作。深化浙广、九广合作，推动更多合作项目落地见效。

五、统筹城乡发展，努力形成协调发展新局面

强化基础设施建设。加大交通基础设施建设投入。加快西成客专建设，实现年底前通车。开工建设广平高速、南山隧道等项目，加强绵万高速广元段招商，升级改造国道212线苍溪至阆中段、亭子口库区环线等国省干线公路和旅游景区公路，新改建县乡公路240公里。启动盘龙机场升级改造，规划建设通用航空体系。开工建设嘉陵江广元段航运二期工程，加快建设广元港张家坝作业区。统筹推进农村“三大建设”，加快推动基础设施和公共服务向农村延伸。推进乐园、曲河、双峡湖、大寨、雷家河等水库和亭子口灌区建设，力争开工建设渔洞河水库。实施农村饮水安全巩固提升工程，新覆盖农村人口10万人。新改建通村公路1260公里，实施渡改桥10座、危桥改造6座。加大新一轮农村电网改造升级力度，加快实施“宽带乡村”工程。扎实抓好省级新农村示范县和市级示范片建设，新建幸福美丽新村250个，创建省级“四好村”160个以上。

深入推进新型城镇化。推进“多规合一”，完成城市总规修编和控规维护，优化“一带一廊两区”空间布局，完善城镇体系。提升中心城区主体功能，开工建设下西大道等项目，提升改造黑石坡森林公园、红军文化园、滨水景观廊道。推进城市修补与生态修复，争取海绵城市和地下综合管廊试点城市建设。抓好白龙水厂建设。加快滨江西路、陵宝二线延伸段等项目建设，提高三江新区综合承载能力。以建设高端服务业聚集区和生态新城为目标，高标准规划建设三江新区宝轮片区。加快东部、北部新城建设，推进昭化、朝天城区和市主城区同城化发展。推动产、城、人、文融合，抓好国家级、省级重点镇和省级、市级统筹城乡发展综合示范镇建设，打造一批特色小镇。强化城市治理，推动城市执法体制改革，推进“四定”网格化管理，加快建设中心城区智慧停车场，提高城市精细化管理和人性化服务水平。

做大做强县域经济。对接国家川陕革命老区振兴发展规划，谋划和推进一批大项目，加快建设川陕甘结合部区域中心城市。坚持错位发展、差异化发展，努力打造各具特色的工业强县、服务业强县、现代农业强县、生态康养旅游强县，改变市县“双弱”局面。启动实施贫困县产业提升行动，大力发展民营经济。理顺县域经济发展管理体制，完善监测和考核指标体系，建立奖惩激励机制。

六、坚持绿色低碳发展，积极推进生态文明建设

加强生态安全屏障建设。扎实开展大规模绿化全川广元行动，全方位推进绿化、彩化、美化、香化、亮化，完成营造林46万亩，增加森林面积5万亩、森林蓄积60万立方米，森林覆盖率提高到56%。推进国家园林城市创建，加快蜀道植物动物园等重点项目建设，实施通道绿化、水系绿化、村庄绿化。加强林业有害生物防治、生物多样性保护和森林防火，大力实施水土保持和生态修复工程。

打好污染防治“三大战役”。着眼建设中国最干净城市，加大工业排放、城市扬尘、秸秆焚烧、生活源污染整治力度，持续推进“无霾城市”建设。全面推行河长制，实行排污许可制，完成市城区19条黑臭水沟整治，确保栖凤湖水环境质量稳步提升。实施嘉陵江、白龙江等主要流域污染防治与生态修复工程，确保水质稳定保持Ⅱ类标准。开展土壤污染防治与修复，建立废弃农膜收集处理体系，推行农用地土壤分类管理。做好迎接全省、全国环保督察工作。深入开展主城区市容环境整治，迎接国家卫生城市复检，推进城乡环境综合治理。

发展低碳循环经济。加快发展清洁能源，支持企业绿色化和园区循环化改造，推广应用装配式建筑。开工建设海螺水泥资源利用项目。加强城镇垃圾、污水处理等基础设施建设，提高垃圾、污水资源化利用率。抓好低碳城市建设，推进公共机构节能。大力发展绿色农业，推进畜禽粪污、病死畜禽、秸秆、废旧农膜及农药包装物等农业废弃物资源化利用，推广生态农业种养模式。

七、着力保障和改善民生，提升人民群众幸福感

集中财力办好民生实事。继续实施十项民生工程，全力办好民生大事，全市一般公共预算支出民生占比保持在65%以上。统筹推进高校毕业生、就业困难人员等重点群体就业，实现城镇新增就业3.2万人。提高社会保障法定人群覆盖率。实施百万安居工程，推进棚户区改造和货币化安置，新开工改造城镇危旧房棚户区3981套。启动实施老旧住宅电梯加装和更新改造。

发展优质教育。实施第三期学前教育行动计划，推进城镇新建小区配套幼儿园移交管理。苍溪县、剑阁县、昭化区、朝天区义务教育均衡发展通过国家督导评估验收。启动“最美乡村学校”创建，推进教师“县管校聘”改革、普职融通试点。改革高中管理体制，全面提高高中阶段教育水平。建设市特殊教育学校。加强职业技能培训，推动农村往届初高中毕业生入读职业学校。

提升医疗卫生服务水平。推进“健康广元”建设，深化医药卫生体制综合改革，加快建设辐射川陕甘毗邻地区的医疗中心。推进与域外高水平医疗机构合作，提高本地医疗机构诊疗水平。深化医疗保险支付方式改革，实施统一的城乡居民基本医疗保险制度，完善分级诊疗制度。加强公共卫生服务，实施全民预防保健行动，推广居民健康卡，推进家庭医生签约服务。实施中医药传承和创新工程，推进中医药强市建设。

繁荣文化体育事业。倡导全民阅读，建设书香社区，广泛开展“百千万”品牌群众文化活动，促进公共文化服务标准化均等化。加强文化遗产保护传承，做好历史文化名城申报工作，办好剑门蜀道国际诗歌节。完善全民健身公共服务体系，积极开展群众体育健身活动，举办市第二届运动会。做好省运会筹备工作。做好审计、统计、住房公积金、老龄、工会、妇女儿童、青少年、慈善、残疾人、红十字会、地方志、档案、外事侨务、精神文明、爱国卫生等工作。

八、加强和创新社会治理，构筑社会治理新格局

完善社会治理。深入推进依法治市，加快法治广元建设。完善社会治理体制，实现政府治理和社会调节、居民自治良性互动。全面开展“七五”普法，深入推进“法律七进”。健全信用体系，加强政务诚信、商务诚信、社会诚信和司法公信建设。完善信访工作制度，健全多元化纠纷解决机制，及时排查化解社会矛盾。加强城乡社区建设，健全基层综合服务管理平台，完善村（居）务公开、厂务公开和民主管理制度。支持群团组织参与社会治理。加强国防动员能力和人民防空体系建设，做好双拥共建、国家安全、保密、民族宗教、对台、防邪等工作。

防范社会风险。深化“平安广元”建设，健全立体化社会治安防控体系，依法严惩违法犯罪活动，严防暴力恐怖事件。全面从严落实安全生产责任，狠抓重点行业领域安全监管，持续开展打非治违和重点专项整治，坚决防范遏制重特大事故。实施最严格的食品药品安全监管，严防严管严控食品安全风险。完善金融风险监测预警和应急处置机制，制止违规违法融资和担保行为，防范化解非法集资、互联网金融等方面风险。做好水文、气象、防震、防汛抗旱、地质灾害防治等工作，加强应急管理，提高防灾减灾能力。

九、加强政府自身建设，不断增强执行力公信力

扎实推进法治政府、高效政府、服务型政府、廉洁政府建设，努力打造人民满意政府。基本健全法治政府的制度机制，提升依法行政能力和水平。坚决执行市人大及其常委会的决议、决定，自觉接受人大法律监督和工作监督、政协民主监督，主动接受社会监督。坚持依法科学民主决策，深化“三同”监督，完善权力运行制约和监督机制。加强和改进执法监督，严格规范公正文明执法。推进“放管服”改革，完善权力清单、责任清单，继续清理规范行政审批和公共服务事项，清理取消涉及群众办事的不合法不合理证明和手续。推行“互联网+政务服务”，强化行政权力网上运行，深化政务公开。加强行风政风建设，优化发展软环境，强化“马上办”意识、群众和企业至上意识、服务意识，坚决整治吃拿卡要、不作为乱作为和庸懒散行为，健全正向激励机制。加强督促检查，狠抓工作落实。严格落实“一岗双责”，把全面从严治党、从严治政各项规定贯彻到政府工作各个环节。加大行政监察力度，严厉查处各类违法违纪案件，对腐败零容忍。

各位代表！时代赋予重任，实干开创未来。让我们更加紧密地团结在以习近平同志为核心的党中央周围，在省委、省政府和市委的坚强领导下，凝心聚力，振奋精神，加快推进整体连片贫困到同步全面小康跨越，以优异的成绩迎接党的十九大和省第十一次党代会胜利召开！
